# Non-Linear Optical Property and Biological Assays of Therapeutic Potentials Under In Vitro Conditions of Pd(II), Ag(I) and Cu(II) Complexes of 5-Diethyl amino-2-({2-[(2-hydroxy-Benzylidene)-amino]-phenylimino}-methyl)-phenol

**DOI:** 10.3390/molecules25215089

**Published:** 2020-11-02

**Authors:** Laila H. Abdel-Rahman, Mohamed S. Adam, Ahmed M. Abu-Dief, Hanan El-Sayed Ahmed, Ayman Nafady

**Affiliations:** 1 Chemistry Department, Faculty of Science, Sohag University, Sohag 82534, Egypt; shakeradam61@yahoo.com (M.S.A.); ahmedabudief@science.sohag.edu.eg (A.M.A.-D.); hanan2013078@science.sohag.edu.eg (H.E.-S.A.); 2 Department of Chemistry, College of Science, King Saud University, Riyadh 11451, Saudi Arabia

**Keywords:** tetradentate, CT-DNA, cytotoxic activity, antimicrobial, NLO

## Abstract

Herein, we report facile procedures for synthesis of a new Schiff base ligand (H_2_L,5-Diethylamino-2-({2-[(2-hydroxy-benzylidene)-amino]-phenylimino}-methyl)-phenol) and its Ag(I), Pd(II) and Cu(II) complexes. The structure of the H_2_L ligand as well as its metal complexes was deduced based on wide range of analytical, structural and spectroscopic tools, along with theoretical evidence via density functional theory (DFT) calculations. The obtained results indicated that the Schiff base (H_2_L) ligand acts as a tetradentate N_2_O_2_ donor with two azomethine nitrogen’s (N1, N2) and two deprotonated phenolic oxygens (O1, O2) atoms. A distorted octahedral structure is assigned to [CuL(OH_2_)_2_]·3/2H_2_O complex and square planar structure for PdL and AgL complexes. The electronic structure and non-linear optical (NLO) property of the prepared compounds were discussed theoretically by the B3LYP/GENECP program. Results revealed that all complexes have non-planner geometries as indicated from the dihedral angles. The charge transfer occurs within the synthesized complexes as indicated from the calculated energy gap between HOMO and LUMO energies. The H_2_L ligand and its complexes are excellent candidates for NLO materials as implied from their hyperpolarizabilities and polarizabilities values. The biological activities of the prepared complexes against selected microorganisms and cancer cell lines gave good growth inhibitory effect. The biocidal potencies of the ligand and its complexes can be arranged as follows: AgL > CuL > PdL > H_2_L, as compared to the used standard drugs. The antiproliferative activity of the studied complexes against different carcinoma cell lines such as liver (Hep-G2), breast (MCF-7) and colon (HCT-116) followed the order H_2_L < AgL< PdL < CuL < vinblastine. Probing the binding interactions of prepared complexes with calf thymus (CT)-DNA using electronic absorption, gel electrophoresis and viscosity measurements revealed strong interaction via intercalation modes, as also evidenced by their molecular docking study.

## 1. Introduction

One of the major health crises of today’s world is antimicrobial drug resistance. To tackle it, there is an immediate requirement for the development of new and much safer drugs and the present work is one such quest for novel and efficient drug candidates.

Schiff base compounds are an important class of ligands that have attracted a great deal of interest owing to their rich coordination chemistry [1,2]. These compounds have extensive applications in different fields. Over the years, these compounds have been used as catalysts, polymer stabilizers and intermediates in organic synthesis, together with their remarkable biological activities, including anticancer, antifungal, antibacterial and antimalarial therapy [3,4].

Tetradentate Schiff base ligands are easily prepared via condensation reaction of 1,2-phenylenediamine or their derivatives with salicylaldehyde. Tetradentate ligands containing N_2_O_2_ have unique catalytic properties and have been used as a catalyst or model system in radio-pharmaceutical and agrochemical industries [5,6,7]. Furthermore, tetradentate metal complexes are widely utilized in drugs’ formulation due to their antimicrobial potency and their facile interactions with DNA and RNA [8]. As for the biological role of selected metals in this study, palladium metal is a suitable candidate for metallodrugs because it displays structural properties similar to those of platinum and exhibits promising in vitro cytotoxicity. Moreover, palladium complexes have significant biological activity such as anti-HIV, antifungal and antitumor activities, and less side effects compared to cisplatin [9,10]. The Cu(II) complexes are favorable candidates for anticancer therapy as reported in conceptually related studies [11]. Silver metal is bioactive even at low concentrations and has a markedly low toxicity. Interestingly, silver complexes with Ag-O and Ag-N bonds showed higher antimicrobial activity than those containing Ag-P and Ag-S bonds [12,13].

Motivated by the promising structural and biological properties of Schiff based metal complexes together with our perpetual engagement in this kind of research endeavor [14,15,16,17,18], here, we offer a facile approach for the synthesis and characterization of a novel tetradentate (N_2_O_2_) Schiff base ligand (H_2_L), (L = 5-Diethylamino-2-({2-[(2-hydroxy-benzylidene)-amino]-phenylimino}-methyl)-phenol), and its metal complexes with Ag(I), Pd(II) and Cu(II) ions. Various spectroscopic, structural and analytical techniques combined with DFT calculations have been employed to deduce the composition, structure and geometry of the prepared complexes. Importantly, the nonlinear optical properties and activity of the complexes were probed by studying the electronic structure. The geometrical parameters, electrostatic potential, natural bonding orbital (NBO) analysis and Quantum global reactivity descriptors of the prepared compounds are calculated by using GENECP/B3LYP. Finally, the antibacterial, antifungal and anticancer activities of the synthesized complexes, together with their interaction with CT-DNA and molecular docking, are extensively investigated. 

## 2. Experimental

Details of the reagents and instrumentation used in this work are shown in the Supplemental Materials.

### 2.1. Formation of the Schiff Base H_2_L Ligand

**Scheme 1:**
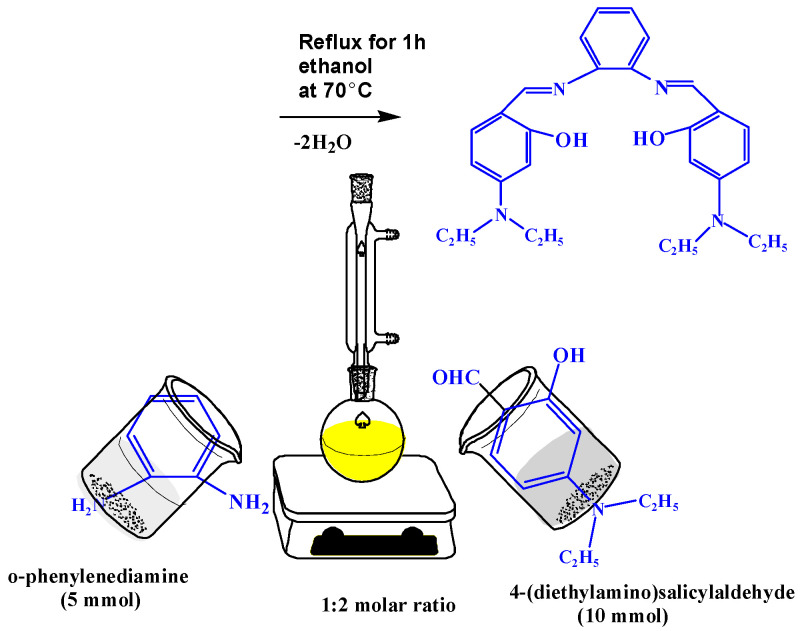
Synthesis of Schiff base ligand H_2_L.

The new Schiff base ligand (H_2_L) was prepared by mixing 4-(diethylamino)salicylaldehyde (10 mmol, 1.93 g) in 20 mL ethanol dropwise with continuous stirring, with 25 mL of ethanolic hot solution of o-phenylenediamine (5 mmol, 0.54 g). The obtained solution was stirred at room temperature for 1 h and then the yellow solid compound was obtained. The solid powder was separated by filtration, washed and dried, as shown in Scheme 1.

Yield 70%; yellow color; melting point (m. p) = 128 °C. FT-IR: 1635 (C=N), 3358 and 3450 for two OH group.^1^H-NMR: 9.62 (s, 2H, 2CH=N), 11.24 (s, 1H, 2OH), 8.57–6.05 (m,10H, =CH_ar_), 3.44–3.38 (q, 8H, 4CH_2_), 1.15 (t, 12H, 4CH_3_).^13^C-NMR: 12.79 (CH_3_), 12.91 (CH_3_), 44.40 (CH_2_), 44.61 (CH_2_), 96.26 (CH), 97.29 (CH), 104.29 (CH), 105.04 (CH), 109.38 (CH),111.5 1(CH), 115.57 (CH), 117.75 (CH), 118.27 (CH), 122.49 (CH), 126.88 (CH), 134.72 (CH), 141.94 (C_q_, CH-N), 151.77 (C_q_, CH-N), 154.39 (C_q_, CH-OH), 160.42 (C_q_, CH-OH), 161.22 (C_q_, CH-CH=N), 163.27 (C_q_, CH-CH=N), 191.32 (2CH=N). Analytical and calculated (Anal. Calc.) for C_28_H_34_N_4_O_2_ (%); C, 73.46; H, 7.50; N, 12.30. Found (%): C, 73.36; H, 7.42; N, 12.22.

### 2.2. Formation of the CuL Complex

The CuL complex was synthesized by adding 25 mL of ethanolic solution of CuCl_2_.2H_2_O (5 mmol, 0.85 g) to 25 mL of ethanolic solution H_2_L ligand (5 mmol, 2.29 g). The obtained solution was stirred for 1 h at room temperature (RT). The precipitate was then separated by filtration, washed and dried over anhydrous CaCl2. Yield 73%; Dark brown color; m. p. = 180 °C. FT-IR: 1635 (C═N) and 3385 and 3418 (-OH). Anal. Calc. for C_28_H_39_N_4_O_5.5_Cu (%): C, 57.55; H, 6.79; N, 9.55. Found (%):C, 57.68; H, 6.69; N, 9.61. Λm: 19.4 Ω^−1^ mol^−1^ cm^2^. μ_eff_: 1.56 B. M.

### 2.3. Synthesis of the PdL Complex

5 mmol of Pd(OAc)_2_ (1.12 g) was dissolved in 25 mL of acetone and added dropwise to 25 mL acetone containing 5 mmol of the H_2_L ligand (2.29 g). The resulting solution was stirred for 2 h under reflux at 50 °C. The crystalline solid compound was then separated by filtration, washed and dried over anhydrous CaCl2.

Yield 76%; Dark brown color; m. p. = 250 °C. FT-IR: 1613 (C=N).^1^H-NMR: 9.62 (s, 2H, 2CH=N), 8.47–5.53 (m, 10H, =CH_ar._), 3.27 (q, 8H, 4CH_2_), 1.31–1.15 (t, 12H, 4CH_3_).Anal. Calc. for C_28_H_32_N_4_O_2_Pd (%): C, 59.84; H, 5.77; N, 9.85. Found (%): C, 59.74; H, 5.68; N, 9.95. Λm: 3.1 Ω^−1^ mol^−1^ cm^2^. μ_eff_ Diamagnetic.

### 2.4. Formation of the AgL Complex

The AgL complex was synthesized by mixing 25 mL of ethanolic solution of AgNO_3_ (5 mmol, 0.849 g) with 25 mL of absolute ethanol solution of H_2_L ligand (5 mmol, 2.29 g). The resulting mixture was heated at 70 °C under stirring for 1 h and left to evaporation overnight. The AgL complex was then separated by filtration, washed, dried and kept in a desiccator. 

Yield 65%; Dark gray color; m. p. > 300 °C FT-IR: 1614 (C=N) and 3385 and 3418 (-OH).^1^H-NMR: 9.60 (s, 2H, 2CH=N), 11.20 (s, 1H, OH), 7.44–6.05 (m, 10H, =CH _ar_), 3.44–3.39 (q, 8H, 4CH_2_), 1.15–1.11(t, 12H, 4CH_3_).Anal. Calc. forC_28_H_33_N_4_O_2_Ag (%): C, 59.56; H, 5.74; N, 10.00. Found (%): C, 59.49; H, 5.84; N, 9.91. Λm: 17.3 Ω^−1^ mol^−1^ cm^2^. μ_eff_: Diamagnetic.

### 2.5. Estimation of the Stoichiometry of the Title Imine Complexes

The simplest spectrophotometric technique adopted to study the equilibria in solutions of complex compounds are the continuous variation and molar ratio methods [16,17,18].

### 2.6. Estimation of Apparent Stability Constant of the Titled Complexes

For the 1:1 complex, the stability constant (*K_f_*) was calculated from spectrophotometric measurements according to the relation presented in our previous work [18,19].

From the stability constants, the change of free energy Δ(G^≠^) was calculated at 25 °C using the relation presented in previous studies [19,20,21].

### 2.7. Kinetic Parameters of the Titled Complexes

The Coats–Red fern relation was used for estimating the kinetic parameters of the titled metal complexes. More details could be found in the previous work [22,23].

### 2.8. Density Functional Theoretical

Density functional theoretical calculations were studied using the Gaussian 09W software package [24,25,26,27,28]. The electronegativity and chemical hardness of prepared complexes was calculated from their highest occupied molecular orbital (HOMO) and lowest unoccupied molecular orbital (LUMO) energy values [29,30]. In this study, the Gauss-view 5.08 visualization program was used for constructing molecular orbitals (MOs) [31]. The hyperpolarizability and polarizability were determined as cited in the literature [32,33,34].

### 2.9. Antimicrobial Activity of the Prepared Compounds

The antimicrobial activity of the new ligand and its metal complexes was screened against strains of Gram (positive) bacteria such as *Staphylococcus aureus* and *Bacillus subtilis,* and Gram (negative) bacteria, such as *Escherichia coli* as well as fungus, namely *Candida albicans*, *Aspergillus flavus* and *Trichophyton Rubrumin*, using the agar well dilution method [19,35,36,37]. The synthesized ligand and its complexes, CuL, PdL and AgL, were dissolved with concentrations of 10 and 20 mg mL^−1^ in dimethyl sulfoxide (DMSO). Nutrient agar and potato dextrose agar were prepared, then sterilized in an autoclave, poured in sterile Petri plates separately and left to cool. Then, the studied bacteria and fungi strains were grown on nutrient agar and potato dextrose agar, respectively. After that, the sterile paper discs of Whatman saturated with the solution of the prepared compounds were placed in the agar using a sterile crook borer for working holes. They were incubated for 24 h at 37 °C for bacteria strains and 72 h at 35 °C for fungi strains. For comparison, the gentamycin drug (standard antibacterial) and fluconazole (standard antifungal) were tested under the same conditions.

### 2.10. CT-DNA Binding Study 

#### 2.10.1. Electronic Spectroscopy 

Electronic spectroscopy was performed by maintaining the titled complexes concentrations and altering the CT-DNA concentration from DNA (0, 10, 20, 30, 40, 50, 60, 70, 80, 90 and 100) mM [19,38,39]. The binding constants (*K_b_*) of the new complexes, have been determined utilizing the spectroscopic titration data applying the following Equation (1):

(1) $$\frac{\left[DNA\right]}{\left(\varepsilon_{a}-\varepsilon_{f}\right)}=\left[DNA\right]\;\frac{1}{\left(\varepsilon_{b}-\varepsilon_{f}\right)}+\frac{1}{K_{b}}\frac{1}{\left[\left(\varepsilon_{b}-\varepsilon_{f}\right)\right]}$$

where *ε_a_*, *ε_f_* and *ε_b_* are the extinction coefficient observed for the charge transfer absorption at a given DNA concentration, the extinction coefficient at the complex free in solution and the extinction coefficient of the complex when fully bound to DNA, respectively. A plot of [*DNA*]\(*ε_a_* − *ε_f_*) versus [DNA] gives *K_b_* as the ratio of the slope to the intercept. The Gibbs-free energy was evaluated from the intrinsic binding constant (*K_b_*) where (Equation (2)):

(2) $$\Delta G_{b}{}^{\neq }=-RTLinK_{b}$$

#### 2.10.2. Dynamic Viscosity Measurements

The viscosity times were determined by keeping the CT-DNA concentration constant (420 μM) with altering the prepared complexes concentrations (0–250 μM) by using an Oswald micro-viscometer at 25 °C. The relative viscosity of binding CT-DNA with the titled complexes was calculated from the relation (Equation (3)):

(3) $$\eta =\frac{\left(t-t{}^{\circ}\right)}{t{}^{\circ}}$$

where *t* is the time for fluidity observed in seconds, *t°* the time for fluidity buffer in seconds and *η*/*η*° (the relative viscosity) was plotted against 1/R, where (Equation (4)):

(4) $$R=\frac{\left[DNA\right]}{\left[complex\right]}$$

#### 2.10.3. Gel Electrophoresis 

The prepared complexes were incubated with CT-DNA for 1 h at 37 °C. Then, the mixtures were mixed with brome phenol blue dye and loaded onto the wells gel (1%) in tris-borate-EDTA (TBE) buffer. Then, a constant voltage (100 V) was applied for 60 min. Finally, a gel image for the binding CT-DNA with synthesized complexes was taken with a Panasonic Lumix DMC-LZ5K 6MP Digital Camera [19].

### 2.11. Molecular Docking Studies

The molecular docking of the prepared complexes was studied by using Dell Precision™ T3600 Workstation and X-ray Crystal Structure of a B-DNA dodecameric (CGCGAATTCGCG)_2_ running 3′–5′ direction at 1.9 Å resolution [19,40].

### 2.12. Cytotoxicity 

The prepared compounds were tested against Hep-G2, HCT-116 and MCF-7 cell lines. The absorbance was measured with an ELISA microplate reader at 564 nm. The inhibitory concentration percent was determined from the following Equation (5) [19,41,42]:

(5) $$IC(\%)=\frac{control_{OD}-compound_{OD}}{control_{OD}}\times 100$$

## 3. Results and Discussion 

### 3.1. H- and ^13^C-NMR Spectra

**Figure 1:**
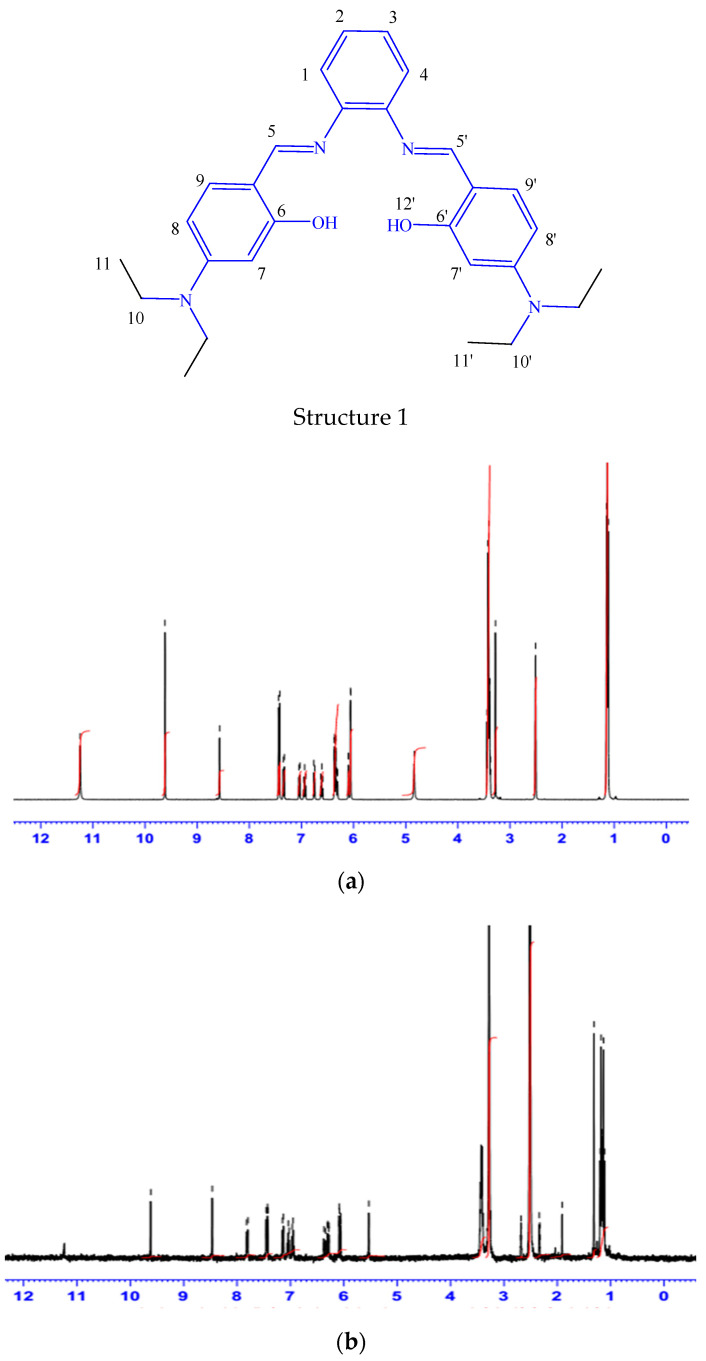
The ^1^ H-NMR spectroscopy of the prepared H_2_L imine ligand (**a**) and its PdL complex (**b**).

The ^1^H-NMR data for the Schiff base was recorded by using DMSO-d6 as a solvent and the chemical shift in ppm. In view of the labelled drawing of H_2_L (Structure 1), the obtained ^1^H-NMR spectra of the ligand and its Pd-complex (Figure 1a,b), the signal of the two azomethine protons (HC=N) of C5 and C5′ appeared at 9.62 ppm, whereas the signal of the two phenolic OH was noted at 11.24 ppm. The spectrum also shows signals of ten aromatic protons in the range of 8.57–6.05 ppm, while the 12 aliphatic protons of (CH3) of C11 and C11′ and the 8 aliphatic protons (CH2) C10 and C10′ were displayed at 1.15 and 2.56 ppm, respectively. Importantly, the higher δ values of the hydroxyl groups are attributed to intermolecular hydrogen bonding [43]. In palladium complex, although most of ligand characteristic peaks are noticeable in the spectrum, the hydroxyl group of the H_2_L ligand (at 11.24 ppm) takes part in complex formation, so it disappears upon complexation, as illustrated in Figure 1b. The ^13^C-NMR spectra of the prepared H_2_L ligand (Supplementary Figure S1) shows a signal at 191.32 ppm, assigned to two azomethine carbons, C5 and C5′. The signals that appeared in 159–112 ppm are consistent with aromatic carbons C1, C2, C3, C4, C7, C8, C9, C7′, C8′ and C9′. The signals that appeared in the region at 12.91–12.79 and 44.61–44.40 ppm are attributed to aliphatic carbons (CH_2_) of C10 and C10′ and (CH_3_) of C11 and C11′, respectively.

### 3.2. Infrared (IR) Spectra

The characteristic IR bands (Supplementary Figures S2 and S3) of the prepared compounds along with their assignments are summarized in Table 1. The structure of the compounds can be easily identified by the presence of both –OH and –CH=N bands in the IR spectra. Thus, the obtained IR spectrum of the Schiff base (H_2_L) ligand displayed a sharp band at 1634 cm^−1^, which could be assigned to C=N stretching of the azomethine group. The shift of this band to a lower wave number (1613–1618 cm^−1^) indicates the involvement of the azomethine group of H_2_L in coordination to the metal ions in all the complexes [44]. The IR spectrum of the ligand also showed a broad band at 3357 cm^−1^ that was attributed to the hydroxyl group. Importantly, in the low-frequency region, new bands observed in the IR spectra of all metal-chelates at 523–528 cm^−1^ are assigned to M–O bond stretch. The other new band at 443–488 cm^−1^ in the infrared spectra of the complexes is for M–N stretching [45]. Taken together, these IR data of the H_2_L ligand and its metal complexes with Pd(II), Cu(II) and Ag(I) clearly indicate that both phenolic oxygen and azomethine nitrogen participated in the chelation with metal ions. Band observed at 3418 cm^−1^ in the IR spectrum of the CuL complex, in particular, may be due to the bending motion of coordinated water molecules.

### 3.3. Evaluation of the Stoichiometry of the New Complexes 

The stoichiometry of complexes under study are present in 1:1 molar ratio of metal to ligand (M:L) as shown in Supplementary Figure S4. Supplementary Figure S5 contains the molar ratio curves, which confirm the same ratio of metal ions to the ligand in the prepared complexes. Importantly, the prepared complexes exhibited high stability, as indicated from the obtained stability constant values. Moreover, the complexation reaction is favored and occurs spontaneously, as evident from the Gibbs-free energy values for the titled complexes in Supplementary Table S1 [22,23].

### 3.4. Elemental Analyses and Conductivity Measurements

Conductivity measurements and elemental analyses of the titled compounds suggested that the prepared H_2_L ligand forms complexes with Pd(II), Cu(II) and Ag(I) ions in 1:1 molar ratio (M:L), as presented in Table 2. Also, all the complexes have a non-electrolytic nature according to the molar conductance values [35,45].

### 3.5. Magnetic Measurements

AgL and PdL complexes are diamagnetic due to their square planar geometry, whereas the CuL complex showed paramagnetic character because of its octahedral geometry, with magnetic moment (μ _eff._) value of 1.56 Bohr Magnetons (B. M.) (Table 2) [46].

### 3.6. Electronic Spectroscopy

**Figure 2:**
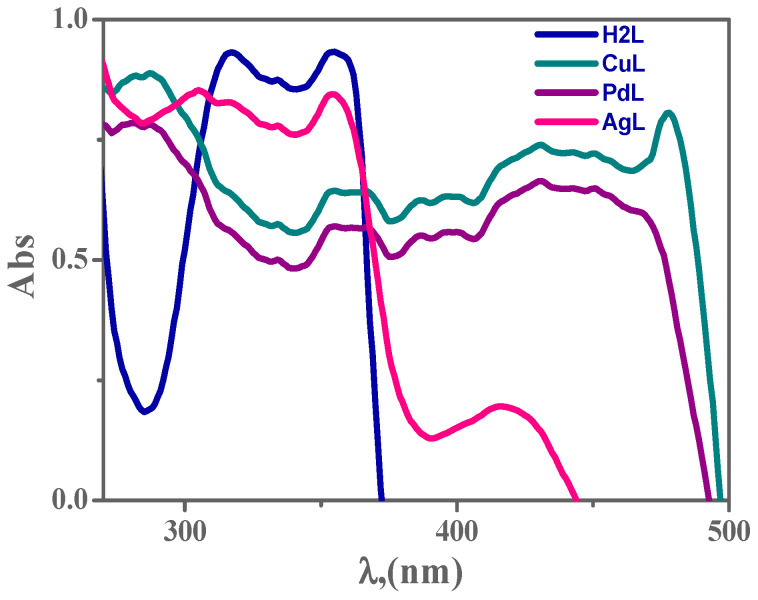
The electronic scans of 1 × 10^−3^ M H_2_L ligand and its metal complexes in dimethylformamide (DMF) at 298 K.

The structural geometry of complexes is confirmed by electronic spectroscopy [41,42,47]. The electronic spectra parameters are presented in Figure 2 and Supplementary Table S2. The *n* → π* transition, forming the imine function group of the H_2_L ligand, appears at ~316 nm. The octahedral complex CuL showed a band at 354 nm due to *n*–π* and a second band at 477 nm assigned to the ligand to metal charge transfer (LMCT), and a band at 560 nm which is attributed to d → d transitions. Moreover, in the electronic spectra of the PdL and AgL complex, two transitions have been observed, the first maxima at 360 and 354 nm respectively, could be assigned to the n–π* transitions of the imine groups and aromatic rings, thus belonging to the intra-ligand charge transfer (ILCT) transitions. The second maxima at 430 and 450 nm for PdL and 417 nm for AgL, are presumably a mixture of ligand-to-metal (LM) and metal-to-ligand (ML) charge transfer (CT) transitions [42]. 

### 3.7. TGA Studies

Thermal gravimetric analysis (TGA) of the prepared complexes was studied using the differential thermogravimetric and the thermogravimetric techniques to calculate the thermal stability and determine water molecules in the complexes structures [41]. The thermograms of the CuL complex show 3/2 H_2_O_hydra_ and 2H_2_O_coord_ molecules in its structure. The thermal behavior of other complexes was tested in the range 25–650 °C in air, recorded in Table 3 and displayed in Supplementary Figures S6 and S7.

#### Kinetic Parameter for Thermal Analysis of the New Complexes 

Kinetic parameters were determined by the Coats Red–fern relation (Table 3). In most thermal steps, the entropy values of the prepared complexes (ΔS^±^) are negative, suggesting the decomposition processes are unfavorable. The enthalpy values of the prepared complexes (ΔH^±^) are positive, thereby showing that decomposition processes are endothermic [15,38]. Negative values for activation entropy of the prepared complexes indicate a huge activated state.

### 3.8. The Stability Range of the New Complexes

The pH-profile contained in Supplementary Figure S8 shows perfect dissociation curves and a high stability over pH range (5–11) of the tested complexes. This behavior highlights the enhanced stability of all prepared complexes thanks to the presence of our novel H_2_L imine ligands. Consequently, the suitable pH domain for the different applications of the tested complexes is from pH = 5 to pH = 11. Additionally, the obtained results also showed that the synthesized complexes are highly stable compared to their corresponding ligand [38]. 

**Scheme 2:**
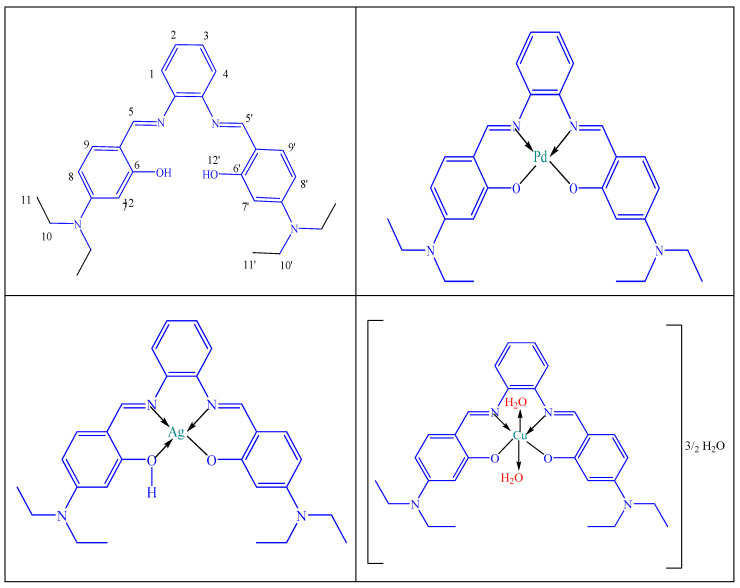
The suggested structures for the H_2_L ligand and its PdL, AgL and CuL complexes.

The proposed structures for the prepared metal complexes are suggested based on IR, electronic spectra, elemental analyses, TGA analysis, magnetic and conductivity measurements, as illustrated in Scheme 2.

### 3.9. MO Calculations

The molecular orbital calculations of the titled compounds were discussed by using B3LYP/GENECP. The studied ions coordinate with the Schiff base via N2, N34, O16 and O45 atoms, forming the 1M:1L complexes.

#### 3.9.1. Geometry of the Ligand 

**Figure 3:**
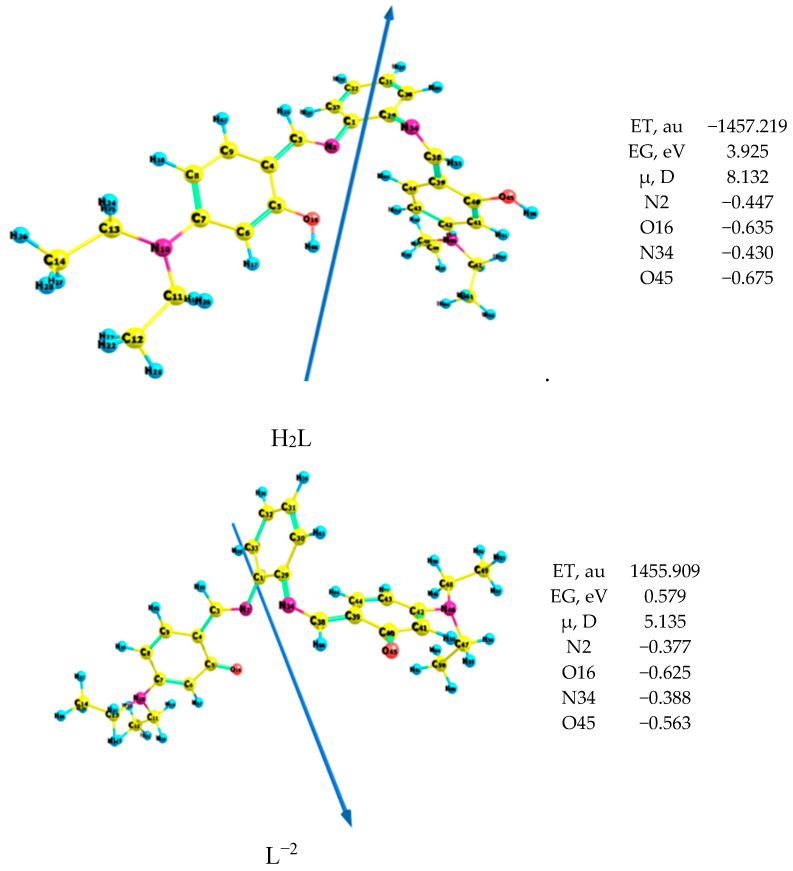
Geometry of the ligand of the H_2_L ligand and its anion L^2−.^

The anion ligand interacts with the studied metal ions to form the complexes because the energy gap of the anion ligand is lower than that of the neutral ligand by 3.35 eV, where the lower the energy gap, the more compound reactivity, as shown in Figure 3. The N2, N34, O16 and O45 atoms in the ligand and its anion coordinate with the metal ions to afford the corresponding complexes, as shown from the natural charges.

#### 3.9.2. Geometry of the Prepared Complexes 

**Figure 4:**
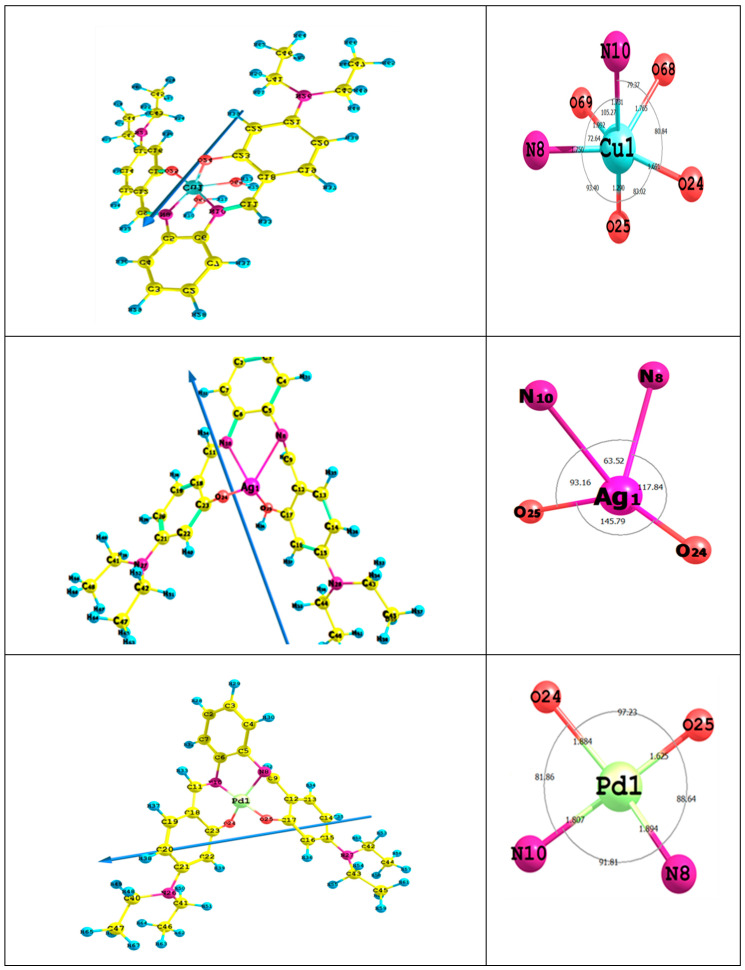
The bond lengths, the dipole moment vector, optimized geometry, numbering system and bond angles for the titled complexes.

Figure 4 presents bond lengths, the dipole moment vector, optimized geometry, numbering system and bond angles of Cu(II)L, Ag(I)L and Pd(II)L complexes. The obtained results revealed a distorted octahedral structure for the Cu complex, whereas both Ag and Pd complexes favored square planar geometry.

*(A) Distorted Octahedral Cu Complex:* In the CuL complex, the Cu(II) ion coordinates to the N10 and O24 atoms, allowing for a six-membered ring (CuO24C23C18C11N10), while with N10 and N8 atoms, a five-membered ring (CuN10C6C5N8) is accomplished. In case of using the N8 and O25 atoms, a six-membered ring (CuN8C9C12C17O25) is favored. The calculated bond angles’ values between the Cu ion and the ligand < O24CuO25 < O25CuN8 < N8CuO69 < O69CuN10 < N10CuO68 and <O68CuO24 are in the range between 72° and 105°, which is assigned to a distorted octahedral complex [48]. The fact that the ligand is not in the same plane of the Cu ion as indicated from the dihedral angles, i.e., <CuO24C23C28 (56°), <CuO25C17C12 (43°), <O68CuN10C6 (142°) and <O68CuN8C5 (119°), shows it is far from 0° or 180°. Most M-O and M-N bond lengths are elongated after complex formation, so they are longer than that of the typical M-X. This behavior indicates that the ionic character of the CuL complex is small [19,49].

*(B) Square Planar PdL and AgL Complexes:* In the PdL complex, the Pd(II) ion coordinate with N8 and O25 atoms, shaping up a six-membered ring (PdN8C9C12C17O25), with N10 and N8 atoms forming a five-membered ring (PdN10C6C5N8) and with N10 and O24 atoms to allow for six-membered ring (PdN10C11C18C23O24). The calculated bond angles values < O25 PdO24, < O24PdN10, < N10PdN8 and <N8PdO25 are in the range between 81° and 97°, which is assigned to a square planar geometry [50,51]. The dihedral angles of the Pd(II) ion < PdO25C17C12 (33°), < PdN8C9C12 (72°), < PdN8C5C6 (17°), < PdN10C6C5 (20°) and < PdO24C23C18 (47°), which are in the range of 0° or 180°, indicated that the ligand is not in the same plane as the Pd(II) ion. In the AgL complex, the Ag-ion coordinates with N8 and O25 atoms, shaping up a six-membered ring (AgN8C9C12C17O25), with N10 and N8 atoms making up a five-membered ring (AgN10C6C5N8) and with N10 and O24 atoms to form a six-membered ring (AgN10C11C18C23O24). The computed dihedral angles showed that the H_2_L ligand is not in the same plane as the Ag(I) ion. The ionic character of PdL and AgL complexes is small due to the enlargement of most M-O and M-N bond lengths compared to that of the typical M-X [52].

#### 3.9.3. Natural Population and Charges

The natural population and charges of the CuL, PdL and AgL complexes are depicted in Supplementary Tables S3 and S4. The N8, O24, N10 and O25 atoms tend to donate electrons to the metal ions, whereas the Cu, Pd and Ag atoms tend to accept electrons from the donating centers. After complexation, the natural charges of the ligand increase and lead to a back donation from the metal ions to the ligand (Supplementary Table S4). The Ag(I), Pd(II) and Cu(II) ions receive 0.2762, 1.4428 and 1.065 electron (e) from the H_2_L ligand, respectively. 

#### 3.9.4. Non-Linear Optical Properties 

The hyperpolarizabilities and polarizabilities of the prepared metal complexes and urea are listed in Supplementary Table S5. The standard prototype used in non-linear optical studies is urea. Urea is a reference in this work where there was no theoretical investigation of non-linear optical properties of the prepared compounds. The analysis of the β parameter showed that the H_2_L ligand is 14 times higher than urea. The β value is 24, 33 and 10 times higher than urea in the PdL, AgL and CuL complexes, respectively. The obtained data implied that the ligand and its complexes are efficient candidates for non-linear optical materials.

#### 3.9.5. Quantum Global Reactivity Descriptors

**Figure 5:**
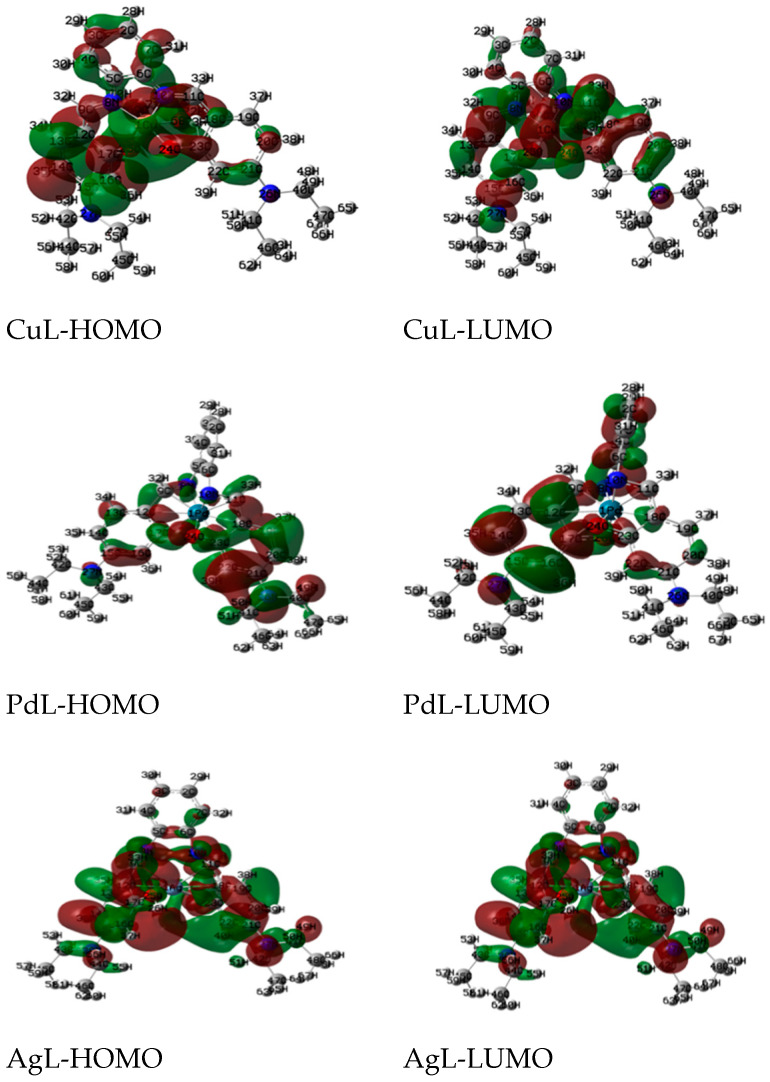
The charge density maps of the highest occupied molecular orbital (HOMO) and lowest unoccupied molecular orbital (LUMO) of studied complexes.

The Quantum global reactivity descriptors of the metal complexes are calculated and displayed in Figure 5. The reactivity of complexes increases in the order: PdL >> Ag L > CuL, thereby indicating that the decrease in the energy gap leads to a softer charge transfer and the polarization carries out in the complex. 

The electronegativity, chemical hardness, potential and softness values are calculated and listed in Table 4**.** The charge transfer within the complexes increases in the order: PdL > AgL > CuL.

### 3.10. Antimicrobial Activity

The prepared H_2_L ligand and its CuL, PdL and AgL complexes were screened against selected bacteria and fungi strains by paper disc plate. The results are reported in Table 5 and Table 6 and shown in Supplementary Figures S9–S12. A comparative study of the antimicrobial activity of the ligand and its metal complexes indicates that complexes exhibit higher potency compared to the free ligand and this enhanced activity is referred to the coordination with metal ions. The enhanced activity of the complexes over the ligand can be explained based on chelation theory [53,54,55]. This enhancement in the activity may be rationalized on the basis that ligands mainly possess the C=N bond, which coordinated to the metal ions. The obtained results revealed that the new complexes have high activity, especially the AgL complex, which has activity very close to the standard drugs gentamycin and Fluconazole. The biocidal activity of the ligand and its complexes can be arranged as follows: AgL > CuL > PdL > H_2_L, as compared to the used standard drugs. Antibacterial bioassay (Table 5) of the ligand and its complexes towards the different strain of Gram-positive ***(+ve)*** and Gram-negative ***(-ve)*** bacteria follows the sequence: *B. subtilis* > *S. aureus* > *E. coli*. Antifungal activity (Table 6) of the compounds follows the order: *C. albicans* > *A. flavus* > *T. rubrum*.

The minimum inhibitory concentration values of the synthesized H_2_L ligand and its CuL, PdL and AgL complexes against the studied strains of fungi and bacteria are summarized in Table 7. The activity index of the synthesized H_2_L ligand and its CuL, PdL and AgL complexes were calculated and depicted in Table 8 according to the following relation (Equation (6)) [19,41,42]:

(6) $$\mathrm{Activity}\;\mathrm{index}\;\left(A\right)=\frac{\mathrm{inhibition}\;\mathrm{zone}\;\mathrm{of}\;\mathrm{complex}\;\left(\mathrm{mm}\right)}{\mathrm{nhibition}\;\mathrm{zone}\;\mathrm{of}\;\mathrm{standard}\;\mathrm{drug}\;\left(\mathrm{mm}\right)\;}\;\times \;100$$

### 3.11. CT-DNA Binding of the New Complexes 

#### 3.11.1. Electronic Spectra of CT-DNA Complex Titration 

**Figure 6:**
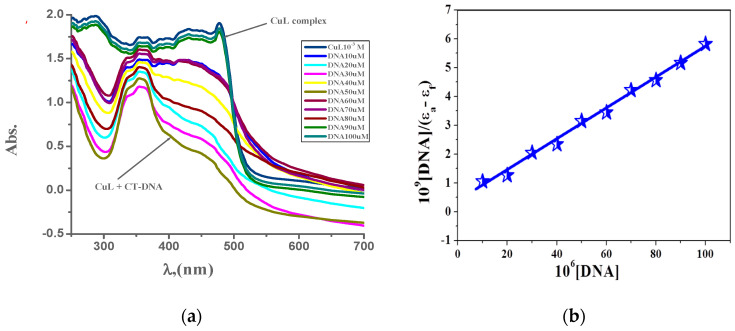
(**a**) Electronic spectroscopy for the interaction of CuL complex (10^−3^ M) with (0–100 μM) CT-DNA in 0.01 M tris buffer at 298 K and (**b**) plot of [DNA]/(ε_a_–ε_b_) versus [DNA] for the interaction CT-DNA.

The absorption spectroscopic technique is a very powerful tool to inspect the mode of interaction of the prepared complexes with CT-DNA, because absorption spectra are very sensitive to changes in the structure of compounds, and the structural change affects the absorption maxima. The electronic absorption spectra of the metal complexes in the absence and the presence of CT-DNA are shown in Figure 6 and Supplementary Figures S13 and S14. Table 9 presents spectral parameters for the interaction of CT-DNA with the studied complexes. Upon gradual addition of CT-DNA, the absorption intensities of ligand-to-metal charge transfer band gradually decreased. As the CT-DNA concentration is increased, a decrease of absorbance for each investigated complex was observed [56,57]. Significant red-shift peak and hypochromicity with increasing the CT-DNA concentrations was detected, which could be referred to stake interaction between the metal complexes and CT-DNA. This behavior suggests that the mode of interaction of CT-DNA with the prepared complexes is intercalation mode. The determined intrinsic binding constants for the new complexes are in the following order: PdL > AgL > CuL [19,27]. The binding constants and spectroscopy parameters of the interaction of the prepared complexes with CT-DNA are illustrated in Supplementary Figures S15–S17.

#### 3.11.2. Dynamic Viscosity Measurement

**Figure 7:**
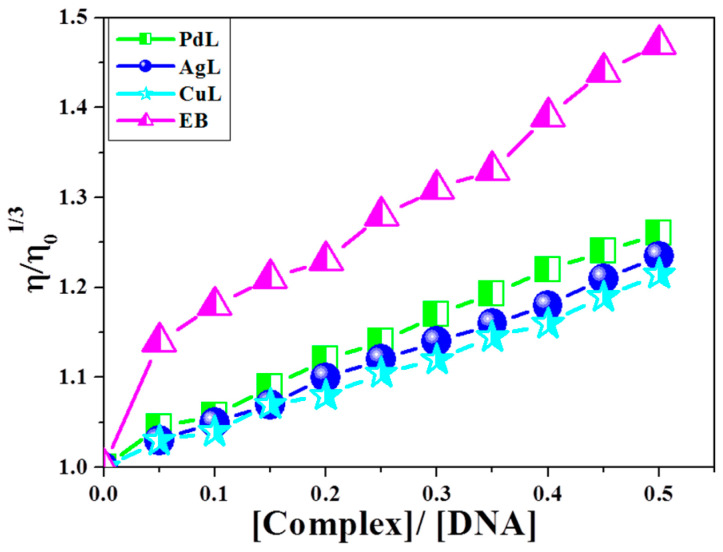
Effect of increasing amount of CuL, PdL and AgL complexes on relative viscosities of CT-DNA at [DNA] = 0.5 mM at 298 K.

Optical spectroscopic tools provide significant, but not sufficient, evidence to support the binding mode for DNA interaction with the studied azomethine complexes. It is known that intercalation and groove binding affect the length of DNA, and hence the viscosity of DNA increases, while electrostatic binding does not affect the length of DNA and hence, its viscosity remains constant [19,56]. Successive addition of complex to a fixed concentration of DNA was undertaken. Upon addition of the new complexes, the relative viscosity of DNA increased as well as the respective complexes (Figure 7). This result is explained on the basis that insertion of complex into the base pairs of DNA increases the length of the DNA chain and consequently, increases the separation of base pairs, which leads to the occurrence of intercalation of compounds into DNA.

#### 3.11.3. Gel Electrophoresis 

**Figure 8:**
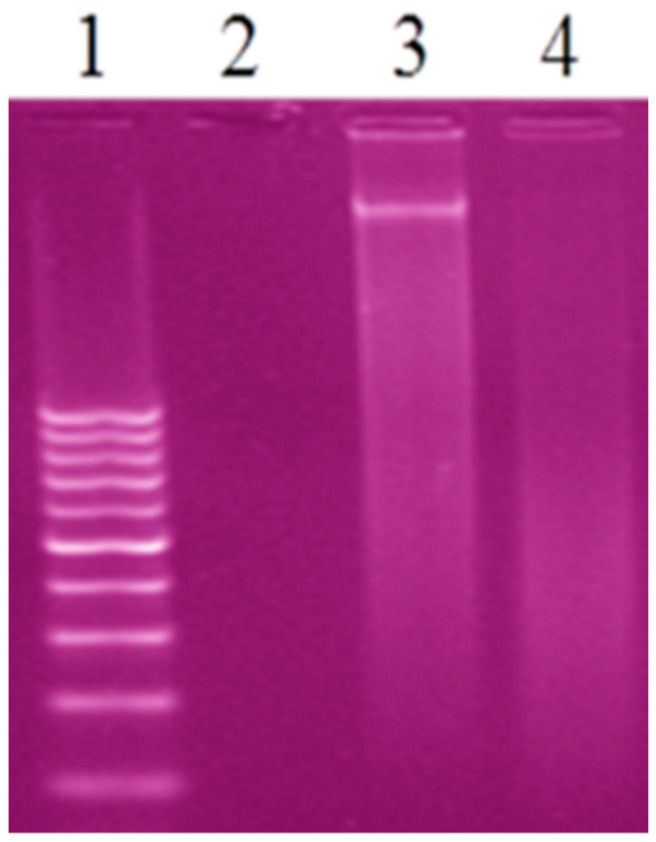
Gel electrophoresis image of the interaction of CT-DNA with the new complexes, Lane 1: 100 DNA ladder, Lane 2: CT-DNA + PdL, Lane 3: CT-DNA + AgL, Lane 4: CT-DNA + CuL.

Agarose gel electrophoresis is the conclusive technique to study the interaction CT-DNA with the prepared complexes. The gel electrophoresis image displayed that the intensity of the CT-DNA loaded with the prepared complexes has partly reduced in AgL and CuL complexes and vanished in the PdL complex because of the cleavage completely for CT-DNA [19,57] (Figure 8). The experimental results suggested that CT-DNA can bind with the prepared complexes via an interactive mode.

#### 3.11.4. Suggested Mechanisms of the Interaction of the New Metal Complexes with DNA

The correlation between hydrodynamic and spectroscopic measurements between the new complexes and DNA may demonstrate several binding modes. The metal chelate interacts with DNA, likely in a mode that includes electrostatic or hydrophobic interaction. As expressed previously, the CuL complex has two replaceable water molecules coordinated to copper(II) ion in solution, which might be substituted with DNA double helix molecules in the solution [35,41,45,58,59]. Likewise, because of the replacement reaction of water ligand molecules from the CuL complex in solution, the explored metal chelates would have a flat part in the middle.

**Scheme 3:**
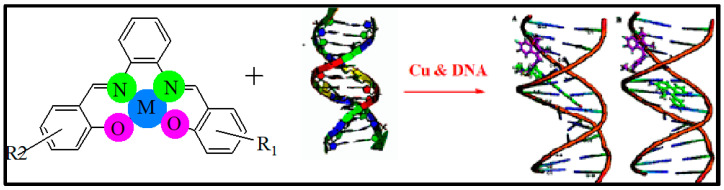
Suggested mechanism for the interaction of CuL complex with DNA via minor groove and intercalation binding.

Accordingly, conceivable interaction of the CuL complex with DNA could be as follows (see Scheme 3): First, interaction of Cu(II) metal chelate with the base backbone of DNA. Second, insertion of the flat part of the metal chelate among the base pairs, and thus coordination of Cu(II) with base pairs of DNA. 

### 3.12. Molecular Docking 

**Figure 9:**
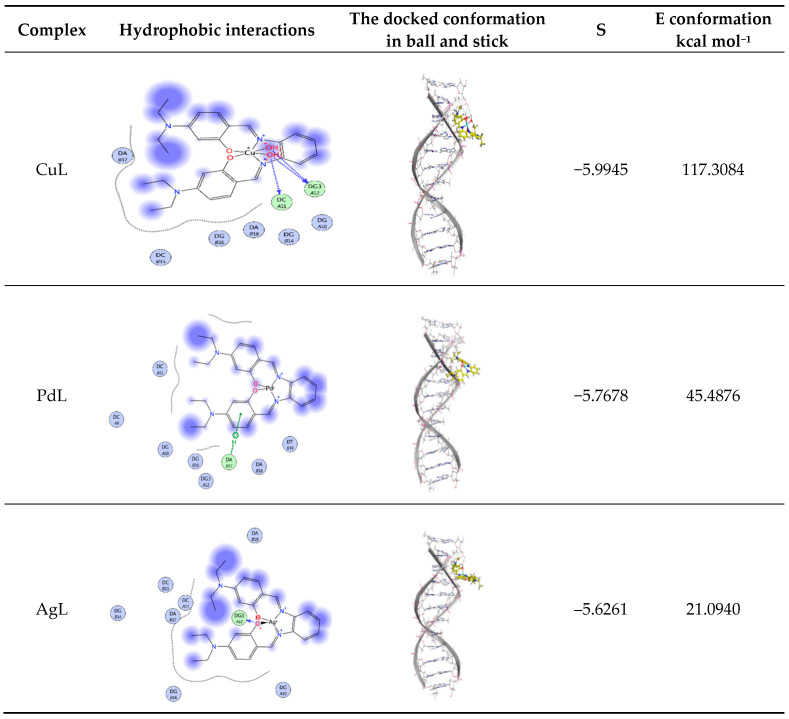
Hydrophobic interaction and docked conformation in ball and stick representation of the interaction of CuL, PdL and AgL complexes with CT-DNA.

Molecular docking aids in understanding the possible mode of interaction and binding affinity of the newly prepared compounds as targeted drug molecules. This most likely gives better insight into how well they exert bioactivity against the target. Therefore, docking studies were performed on B-DNA (PDB ID:1BNA) in the presence of Cu(II), Pd(II) and Ag(I) complexes. The 2D and 3D binding sites scheme of the PdL, AgL and CuL complexes with B-DNA (PDB ID:1BNA) (1T46) is presented in Figure 9.

Docking study results revealed that the CuL complex interacted with DNA, giving the best binding score of −6.0040 owing to the formation of three hydrogen bonds with the amino acids residues DC A11 and DG A12, along with the two coordinated water molecules of the complex besides the hydrophobic interactions between the three phenyl rings of the aromatic system and the ethyl groups of the side chain with the residues DA B17 in the active cavity. The total energy of conformation is −955.3530 kcal mol^−1^_._

The PdL complex showed π–H interaction of the phenyl ring of the Schiff base ligand with DA B17, and hydrophobic interactions between the three phenyl rings of the aromatic system. Additionally, the ethyl groups of the side chain with the different residues such as DC A11 and DG B16 were observed. The binding score is −5.7678 and the energy conformation is −45.4876 kcal mol^−1^ (Figure 9). One hydrogen bond was formed between the phenolic oxygen of the ligand and the amino acid residue DG3 A12 for the AgL complex in addition to the hydrophobic interactions between the three phenyl rings and the ethyl groups with the amino acid residues. The lowest value of the binding score of –5.6261 and conformation energy of 21.0940 kcal mol^−1^ were detected. The docking studies showed that the prepared docked complexes fit mainly in the DNA minor groove and involve hydrophobic as well as hydrogen bonding interactions with DNA bases. Most of the optimal results of docking were in the GC region.

### 3.13. In Vitro Cytotoxicity

**Figure 10:**
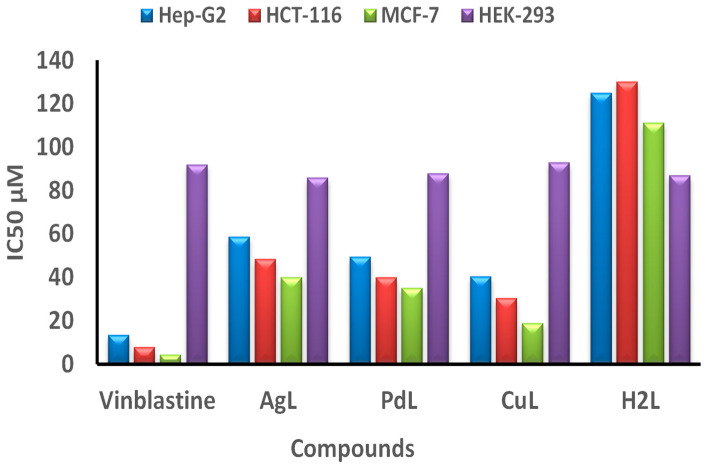
IC_50_ values in μM of the H_2_L ligand, and its CuL, PdL and AgL complexes compared to vinblastine against HCT-116, Hep-G2, MCF-7 and HEK-293 cell lines.

The anticancer activity of H_2_L Schiff base ligand and its CuL, PdL and AgL against HCT-116, HepG-2 and MCF-7 cells was evaluated using the MTT assay. Cell viability curves were plotted and the results were expressed in terms of the minimum inhibition concentration (IC_50_) values as shown in Figure 10 and Supplementary Table S6. IC_50_ (Supplementary Table S6) showed that the cytotoxic efficacy of the prepared metal complexes follows the order: Vinblastine > CuL > PdL> AgL > H_2_L ligand. From the results, it is evident that the CuL complex exhibited higher in vitro cytotoxicity against all selected cell lines when compared to the ligand, as well as to the standard drug, vinblastine. In contrast, the AgL complex showed a lower anticancer activity when compared to the standard drug. It is generally thought that the cytotoxicity of metal chelates depends on their ability to bind DNA and thus damage its structure, which is followed by inhibition of replication and transcription processes and eventually cell death [19,58,59]. Thus, the higher cytotoxicity exhibited by the CuL complex may be attributed to the stronger binding ability of that complex with DNA, as discussed above.

The tested compounds exhibited good anticancer activity against hepatocellular carcinoma (Hep-G2 cell line) with IC_50_ values of 30.2–48.5 μM. The least activity was observed for colon carcinoma (HCT-116) cell lines, which gave IC_50_ values from 40.4 to 58.8 μM. The cytotoxic activity of the H_2_L ligand and its compounds against breast carcinoma (MCF-7 cell line) was in the range of IC_50_ values from 18.9 to 40.1 μM, which is the best compared to the other cell lines. The cytotoxic efficacy of the compounds against the different carcinoma cell lines (Hep-G2 cell line), breast carcinoma (MCF-7 cell line) and colon carcinoma (HCT-116 cell line) follows the order: MCF-7 > Hep-G2 > HCT-116. 

Moreover, the CuL complex showed the most powerful anticancer effect with an IC_50_ value of 18.9 μM against the breast carcinoma (MCF-7) cell line, which is relatively comparable to that of the reference drug vinblastine (IC_50_ = 4.44 μM), as shown in Supplementary Table S6. More importantly, the prepared metal complexes are highly specific towards cancer cell lines only, as they exhibited mild toxicity against normal cell line (HEK-293), as illustrated in Figure 10. Taken together, the combined cytotoxicity results of the new complexes, supported by the data obtained from the protein interaction, revealed that the CuL complex has the best binding score and conformation energy value, whereas AgL showed the lowest binding score to DNA.

## 4. Summary and Conclusions

Three new complexes of the Schiff base (H_2_L) ligand, namely CuL, AgL and PdL, were synthesized and fully characterized using numerous physicochemical and spectral tools. The obtained results showed that the ligand H_2_L acts as a bi-negative tetradentate (NNOO) manner around the Pd(II), Cu(II) and Ag(I) ions. From the analytical data, PdL and AgL complexes have been assigned square planar geometry, while CuL has distorted octahedral geometry. In all complexes, most M-O and M-N bond lengths show extending after complex formation, and this indicated that the ionic character of the complexes is small. The β value was 24, 33 and 10 times higher than urea in the PdL, AgL and CuL complexes, respectively. These results clearly established that the ligand and its complexes are efficient candidates for non-linear optical materials. The antipathogenic activities showed that the prepared complexes have promising bioactivities against the tested microorganisms as compared to standard drugs. Particularly, the AgL complex could effectively inhibit the growth of all the tested microorganisms, with minimum inhibitory concentration (MIC) values in the range 0.75–2.00 μM. The CT-DNA interaction mode with the prepared complexes is intercalative mode. The molecular docking indicated that the title complexes are appropriate mainly in the minor groove of CT-DNA and involve hydrogen bonding and hydrophobic interactions with DNA bases. Furthermore, cytotoxic results showed that all prepared complexes display good bioactivities against the selected cancer cells lines as compared to reference drugs. The antiproliferative activity of the studied complexes against Hep-G2, HCT-116 and MCF-7 cell lines increased in the following series: H_2_L < AgL< PdL < CuL < vinblastine. Especially, the CuL complex showed the best inhibition growth against the MCF-7 cell line with the IC_50_ value equal to 18.9 μg/mL (μM) compared to vinblastine. Finally, the significant binding energies obtained from the molecular docking study suggested that the proposed Cu(II), Pd(II) and Ag(I) metal complexes are more potent bio compounds compared to the parent H_2_L ligand. This means that the therapeutic properties of the organic molecules could be dramatically improved upon complexation with desired metal ions. 

## Figures and Tables

**Table 1:** The characteristic IR bands (cm^−1^) of the synthesized compounds.

Compounds	υ(OH)/H_2_O	υ CH) _ar_	υ (C=N)	υ (C-O)	υ (M-O)	υ (M-N)
H_2_L	3357	3044	1635	1279	---	---
CuL	3418	3061	1618	1252	524	443
PdL	---	3056	1613	1244	523	444
AgL	3437	3069	1624	1241	528	488

**Table 2:** Physicochemical and analytical data of the new titled compounds.

Compound Molecular Formula	M. p. (°C)	Molecular Weight	Color	Λ_m_ (Ω^−1^cm^2^ Mol^−1^)	μ_eff._ (B. M.)	Analysis (%) Found (calc.)		
H_2_L C_28_H_34_N_4_O_2_	124	458.0	yellow	---	---	73.46 (73.36)	7.50 (7.42)	12.30 (12.22)
[CuL(OH_2_)_2_].3/2H_2_O C_28_H_39_N_4_O_5.5_Cu	180	582.5	Dark brown	19.4	1.56	57.55 (57.68)	6.79 (6.69)	9.55 (9.61)
PdL C_28_H_32_N_4_O_2_Pd	250	562.4	Dark brown	3.1	Diamagnetic	59.84 (59.74)	5.77 (5.68)	9.85 (9.95)
AgL C_28_H_33_N_4_O_2_Ag	< 300	564.8	Dark gray	17.3	Diamagnetic	59.56 (59.49)	5.74 (5.84)	10.00 (9.91)

**Table 3:** TGA and the kinetic parameters of the prepared complexes.

Complexes	Dec. Product	Dec. Temp (°C)	Weight Loss %		E^±^ (kJ Mol^−1^)	A × 10^4^ (s^−1^)	ΔS^±^ (J Mol^−1^)	ΔH^±^ (kJ Mol^−1^)	ΔG^±^ (kJ Mol^−1^K^−1^)
			Found	Calc.					
CuL	3/2 H_2_O	25–100	4.72	4.63	13.00	4.60	−17.63	12.17	13.93
	2 H_2_O	100–218	6.28	6.18			−18.41	11.19	15.20
	C_11_H_14_N_2_	220–350	29.97	29.87			−18.88	10.09	16.70
	C_11_H_14_N_2_O	350–420	32.51	32.62			−19.07	9.51	17.52
	C_6_H_4_	425–625	12.97	13.05			−19.46	7.80	19.96
Residue	CuO	<625	13.55	13.65			---	---	---
PdL	C_10_H_13_N	30–260	26.02	26.14	29.00	2.8	−19.08	26.84	31.80
	C_11_H_14_NO	260–410	31.19	31.30			−19.54	25.59	33.60
	C_7_H_5_N_2_	410–600	20.92	20.80			−19.92	24.01	35.96
Residue	PdO	<605	21.87	21.76			---	---	---
AgL	C_11_H_14_N_2_O	37–270	33.54	33.64	27.00	2.6	−19.19	24.75	29.93
	C_11_H_15_N_2_O	274–425	33.91	33.81			−19.65	23.47	31.82
	C_6_H_4_	430–605	13.55	13.45			−20.00	21.97	34.07
Residue	1/2Ag_2_O	<610	20.40	20.50			---	---	---

**Table 4:** Quantum and non-quantum global properties of the studied compounds.

Parameters	H_2_L	PdL	AgL	CuL
E_T (_a.u._)_	−1457.219	−2247.595	−2395.74	−2220.60
E_HOMO_ a. u.	−0.1778	−0.1598	−0.1854	−0.1199
E_LUMO_ a. u.	−0.0336	−0.1176	−0.1282	−0.0428
E_g_, eV	3.9222	1.1478	1.5558	2.0971
I, eV	4.8361	4.3465	5.0428	3.2368
A, eV	0.9139	3.1987	3.4870	1.1641
χ, eV	2.8750	3.7590	4.2649	2.2004
ŋ, eV	1.9611	0.5603	0.7779	1.0363
S, eV^−1^	0.2549	0.8924	0.6427	0.4825
V, eV	−2.8750	−3.7590	−4.2649	−2.2004

**Table 5:** Antibacterial bioassay results of the prepared compounds against studied strains of bacteria compared to gentamycin.

	Inhibition Zone (mm)					
*Compounds*	*E. coli (-ve)*		*B. subtilis (+ve)*		*S. aureus (+ve)*	
Concentration (mg/μL)	10	20	10	20	10	20
H_2_L	5 ± 0.33	9 ± 0.15	7 ± 0.17	15 ± 0.21	6 ± 0.33	13 ± 0.53
CuL	16 ± 0.14	33 ± 0.54	19 ± 0.09	43 ± 0.17	17 ± 0.52	39 ± 0.23
PdL	15 ± 0.27	31 ± 0.26	18 ± 0.09	39 ± 0.12	16 ± 0.17	36 ± 0.19
AgL	19 ± 0.292	36 ± 0.19	23 ± 0.19	48 ± 0.54	20 ± 0.41	42 ± 0.11
Gentamycin	21 ± 0.81	41 ± 0.43	27 ± 0.25	52 ± 0.82	26 ± 0.93	46 ± 0.27

**Table 6:** Antifungal bioassay results of the prepared compounds against studied strains of fungi compared to fluconazole.

*Compounds*	Inhibition Zone (mm)					
	*C. albicans*		*A. flavus*		*T. rubrum*	
Concentration (mg/μL)	10	20	10	20	10	20
H_2_L	6 ± 0.15	10 ± 0.15	5 ± 0.18	9 ± 0.61	4 ± 0.15	7 ± 0.14
CuL	18 ± 0.32	30 ± 0.18	16 ± 0.42	27 ± 0.11	14 ± 0.31	24 ± 0.53
PdL	16 ± 0.13	27 ± 0.34	14 ± 0.15	25 ± 0.19	9 ± 0.42	20 ± 0.51
AgL	22 ± 0.45	35 ± 0.35	18 ± 0.35	30 ± 0.29	16 ± 0.22	25 ± 0.40
Fluconazole	23 ± 0.45	36 ± 0.52	18 ± 0.59	33 ± 0.78	14 ± 0.81	25 ± 0.80

**Table 7:** The minimum inhibitory concentration values of the new H_2_L ligand and its CuL, PdL and AgL complexes against the selected strains of bacteria and fungi.

Compound	The Minimum Inhibitory Concentrations (mg/mL)					
	*E. coli (-ve)*	*B. subtilis (+ve)*	*S. aureus(+ve)*	*C. albicans*	*A. flavus*	*T. rubrum*
H_2_L	7.50	6.00	5.75	5.75	5.50	7.25
CuL	2.75	2.00	2.50	1.75	2.25	2.75
PdL	3.25	3.00	3.00	2.75	2.75	3.00
AgL	2.00	1.00	1.50	0.75	1.25	1.75
Gentamycin	1.25	0.75	1.00	-	-	-
Fluconazole				0.50	0.85	1.05
Control (DMSO)	0.00	0.00	0.00	0.00	0.00	0.00

**Table 8:** The activity index of the new H_2_L ligand and its CuL, PdL and AgL complexes against selected strains of bacteria and fungi.

	The Activity Index (%)					
Compound	*E. coli*	*B. subtilis*	*S. aureus*	*C. albicans*	*A. flavus*	*T. rubrum*
H_2_L	22.5	29.41	28.88	27.02	28.12	26.92
CuL	82.5	84.31	86.66	81.08	84.37	76.90
PdL	77.5	76.47	80.00	72.92	78.12	92.30
AgL	90	94.11	93.33	94.59	93.75	

**Table 9:** Spectroscopic parameters and binding constant results of the CT-DNA reaction with the new complexes.

Complex	λ_max_ Free (nm)	λ_max_ Bound (nm)	Type of Chromism	∆ n	Chromism (%) ^a^	Binding Constant × 10^5^	∆G k J mol^−1^
CuL	239	240	Hypo	1	7.25	1.20	−28.97
	363	357		−6	8.05		
PdL	239	240	Hypo	1	8.51	1.73	−29.88
	358	357		−1	9.47		
	252	251		−1	10.94		
AgL	239	238	Hypo	−1	11.92	1.54	−29.59
	355	356		1	11.49		

## Supplementary Materials

The following are available online at, Figures S1 and S2 ^13^C NMR and IR spectra of H_2_L ligand, Figure S3 IR of AgL complex, Figures S4 and S5 continuous variation and molar ratio plots, Figures S6–S8 TGA and pH profile, Figures S9-S12 antibacterial, antifungal and antivirus activities, and Figures S13–17 electronic absorption and the CT-DNA interaction for prepared ML complexes. Tables S1–S6: formation constants, electronic spectral measurements, neutral charge, electronic configuration, hyperpolarizabilities and Cytotoxic activity.
