# Supplementary material for: Non-Linear Optical Property and Biological Assays of Therapeutic Potentials Under In Vitro Conditions of Pd(II), Ag(I) and Cu(II) Complexes of 5-Diethyl amino-2-({2-[(2-hydroxy-Benzylidene)-amino]-phenylimino}-methyl)-phenol
Source: Molecules. 2020 Nov 2;25(21):5089. doi: 10.3390/molecules25215089 (PMC7662626; doi:10.3390/molecules25215089)
Supplement: Supplementary file 1 [file molecules-25-05089-s001.pdf]

Non Linear Optical Property and Biological Assays of Therapeutic Potentials under in vitro Conditions of Pd(II), Ag(I) and Cu(II) complexes of 5-Diethyl amino-2-({2-[(2-hydroxy-benzylidene)-amino]-phenylimino}-methyl)-phenol

Laila H. Abdel-Rahman[1*], Mohamed Shaker S. Adam[1], Ahmed M. Abu-Dief[1], Hanan El-Sayed Ahmed[1], and Ayman Nafady [2*]

[1]*Chemistry Department, Faculty of Science, Sohag University, Sohag82534, Egypt*

[2]*Department of Chemistry, College of Science, King Saud University, Riyadh-11451, Saudi Arabia*

S1. Material and instrumentation

o-phenylenediamine and 4-(diethylamino) salicylaldehyde, the metal salts ($CuCl_2.2H_2O$, $Pd(OAc)_2$, and $AgNO_3$) and Calf-thymus DNA (CT-DNA) were used for our study and purchased from Sigma–Aldrich (Germany). TBE Buffer (Tris-borate-EDTA) (50X), top vision agarose, ethidium bromide solution (10 mg/mL), 6X DNA Loading Dye and 100 bp DNA Ladder are utilized in gel electrophoresis and purchased from Thermo Fisher Scientific. The synthesized compounds were characterized by measuring melting points by using a melting point device (Gallenkamp, UK), IR spectra by using a Shimadzu FTIR-8300 spectrophotometer, ^1H and ^{13}C NMR spectra by using a Bruker Advance DPX-500 spectrometer where the synthesized compounds were dissolved in deuterated dimethyl sulfoxide (DMSO) solution. All of the scanning UV–visible spectra in DMF were recorded using Quawell UV-vis spectrophotometer 5000. Elemental analysis was conducted on an elemental analyzer (PerkinElmer 240c) at the main laboratory of Cairo University. Thermogravimetric analysis was conducted with a heating rate 5 °C min^{-1} with DTG 60H Detector. Magnetic susceptibility was measured using a Gouy balance and diamagnetic corrections were executed by Pascal's constants. Molar conductivity measurements were performed utilizing a JENWAY 4510 conductivity meter. The values of absorbance of each complex were determined at various pH levels. The pH levels were calculated utilizing a series of Britton universal buffers. The pH values were measured by using An ADWA AD1020 and AD1000 pH meter at 298 K that it is equipped with a CL-51B combined electrode and calibrated with standard buffers (pH 4.02 and 9.18) before measurements.

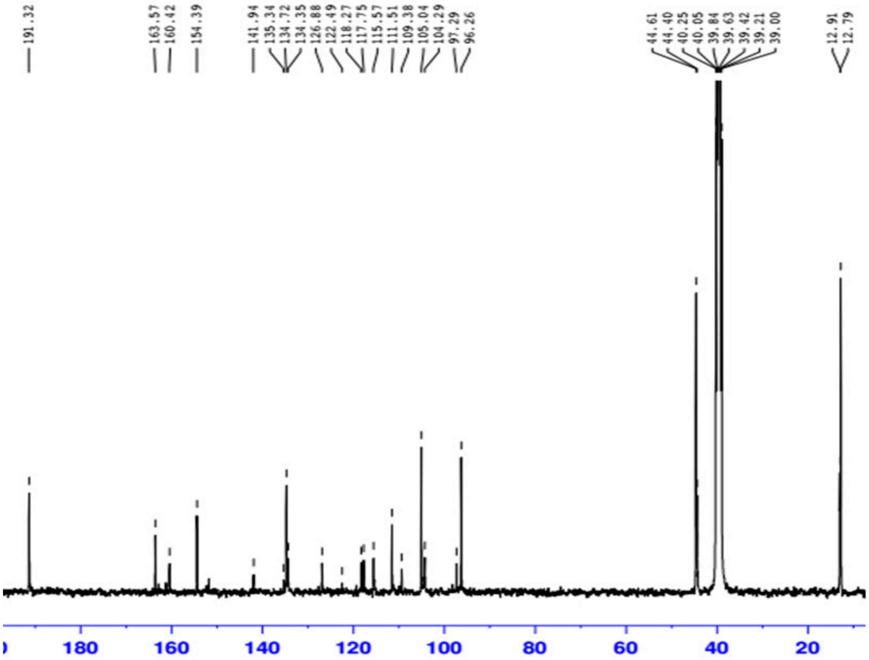

Figure S1: ^{13}C-NMR spectroscopy of prepared H_2L imine ligand.

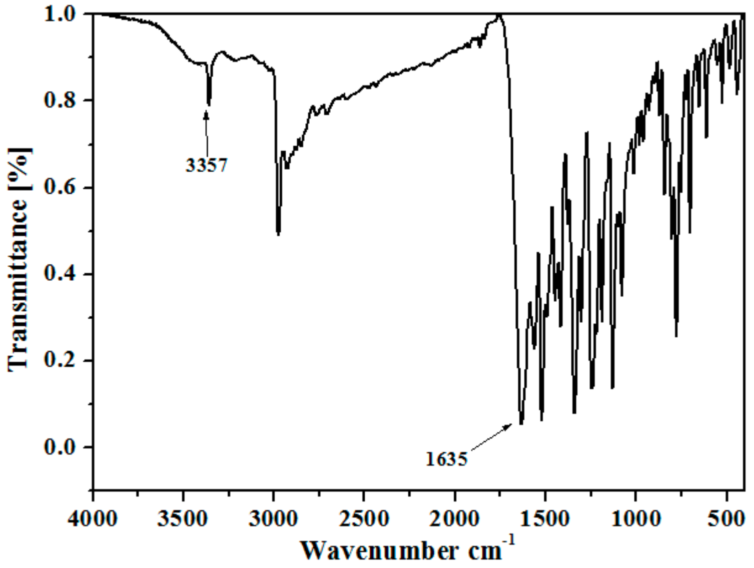

Figure S2: IR spectroscopy of prepared H_2L imine ligand.

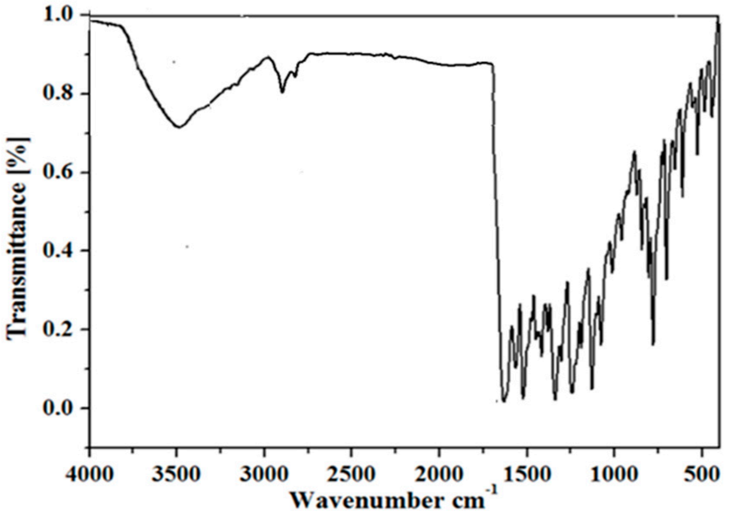

Figure S3: IR spectroscopy of prepared AgL complex.

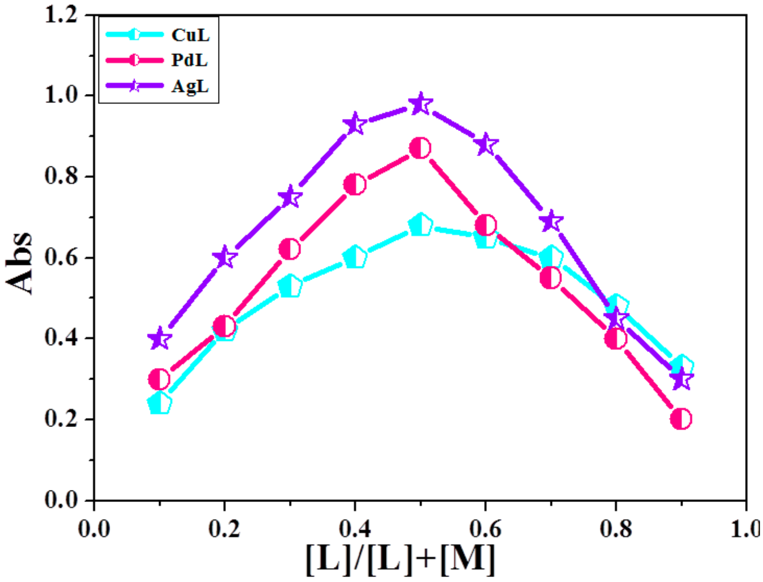

Figure S4: The curve of continues variation method of the prepared complexes in aqueous-ethanol medium at [M] = [H_2L] = 10^{-3} M at 298 K

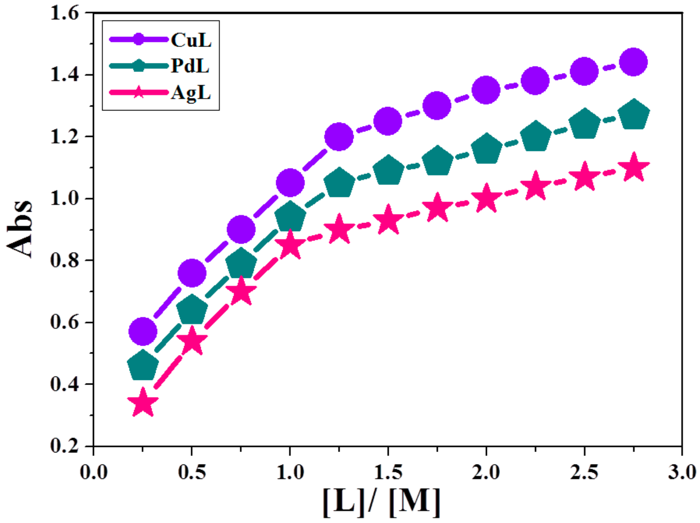

Figure S5: The curve of molar ratio method of the prepared complexes in aqueous-ethanol medium at [M] = [H_2L] = 10^{-3} M at 298 K.

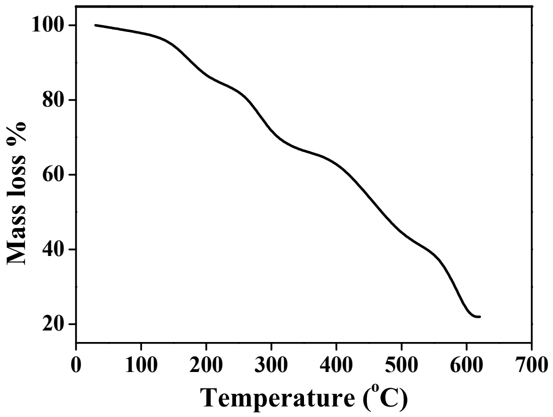

Figure S6: TGA curve of the studied PdL complex.

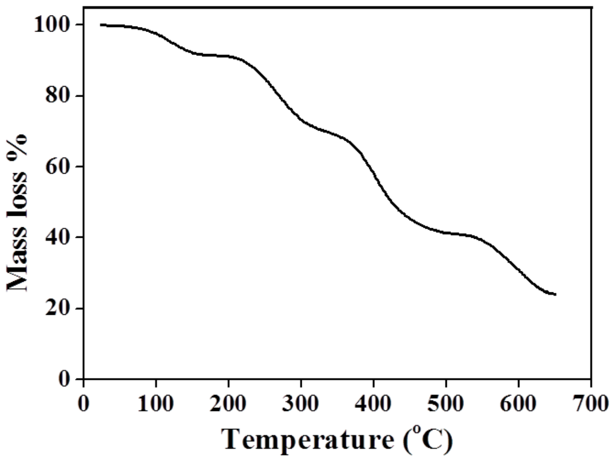

Figure S7**:** TGA of the new CuL complex at heating rate 5 ℃ / min to 650 ℃ in air.

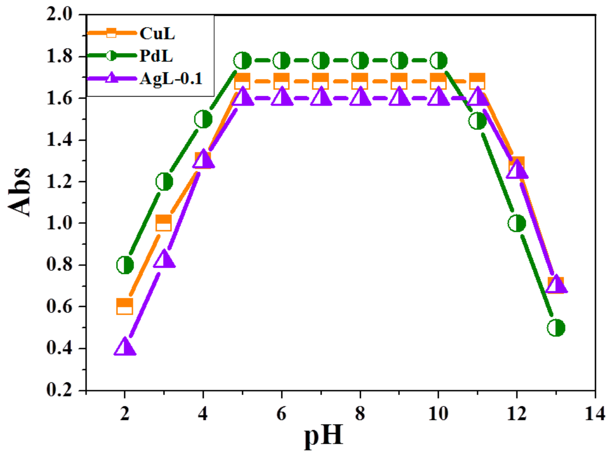

Figure S8: The curve of the pH and absorbance of the new complexes in DMF at 298 K.

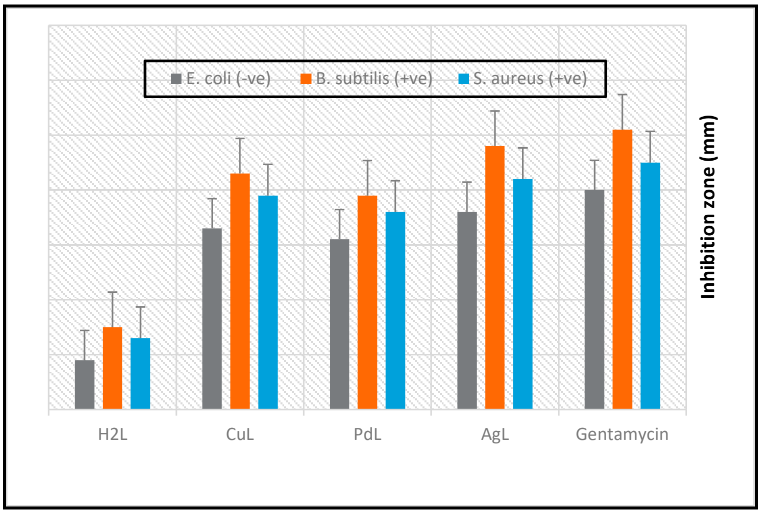

Figure S9. Antibacterial bioassay results of the new H_2L ligand, its CuL, PdL and AgL complexes and Gentamycin with concentration 20 μM.

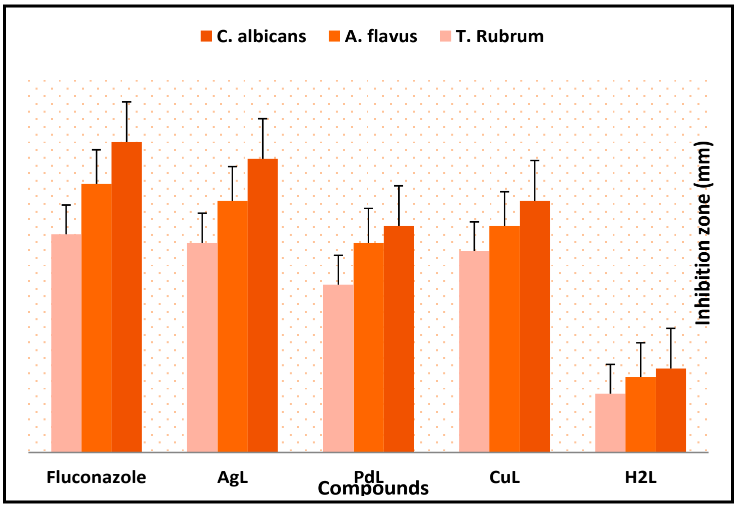

Figure S10. Antifungal bioassay results of the new H_2L ligand, its CuL, PdL and AgL complexes and Fluconazole with concentration 20 μM.

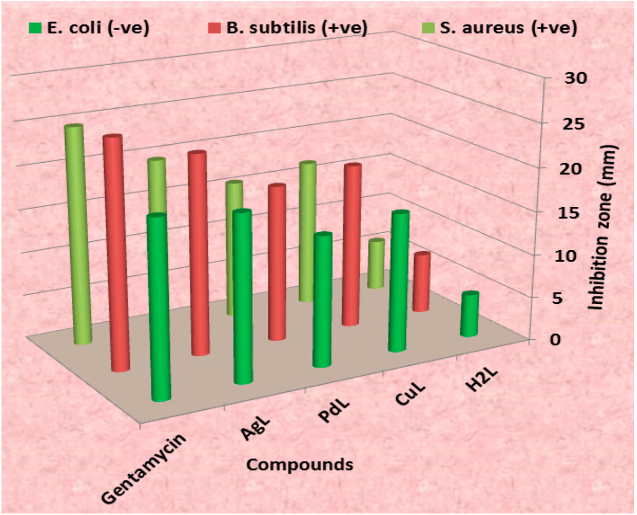

Figure S11: Histogram showing the comparative antibacterial activities of prepared H_2L ligand and its imine complexes (CuL, PdL and AgL complexes) with concentration 10 mg ml^{-1}.

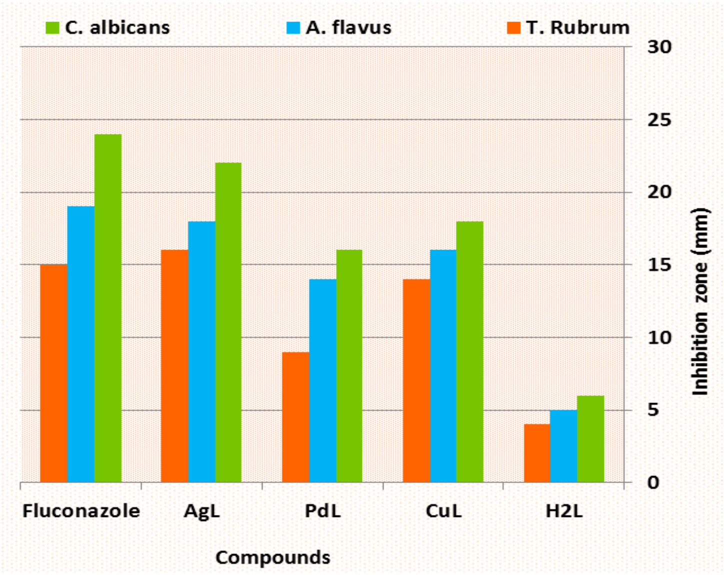

Figure S12: Histogram showing the comparative antifungal activities of prepared H_2L ligand and its imine complexes (CuL, PdL and AgL complexes) with concentration 10 mg ml^{-1}.

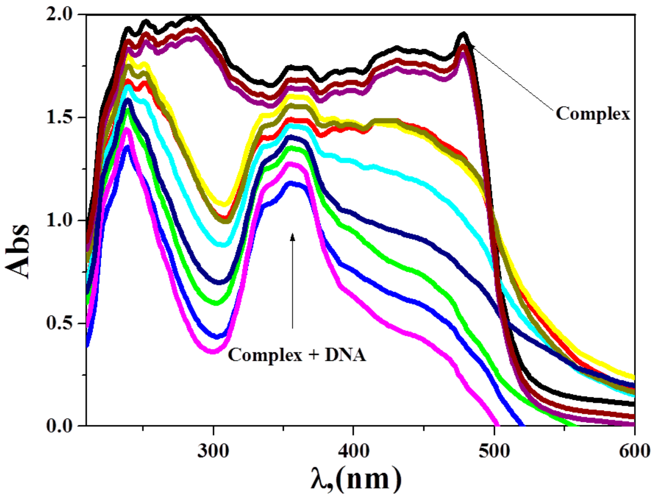

Figure S13: Electronic absorption scans for binding CT-DNA with PdL complex (10^{-3} M) in 0.01 M tris buffer (pH = 7.2, 298 K) with CT-DNA (0 – 100 μM).

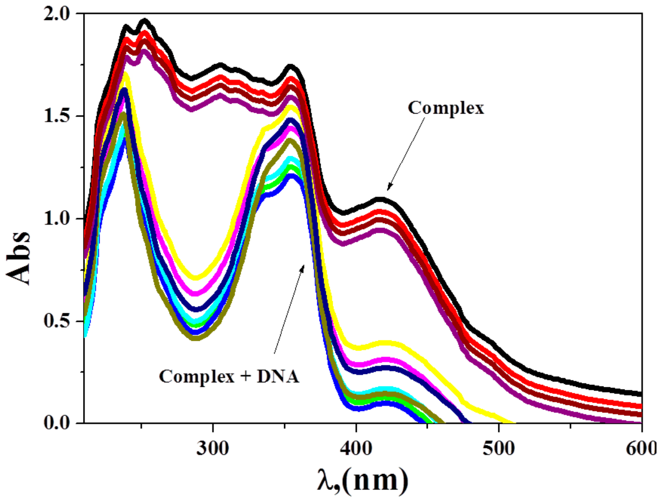

Figure S14: Electronic absorption scans for binding CT-DNA with AgL complex (10^{-3} M) in 0.01 M tris buffer (pH = 7.2, 298 K) with CT-DNA (0 – 100 μM).

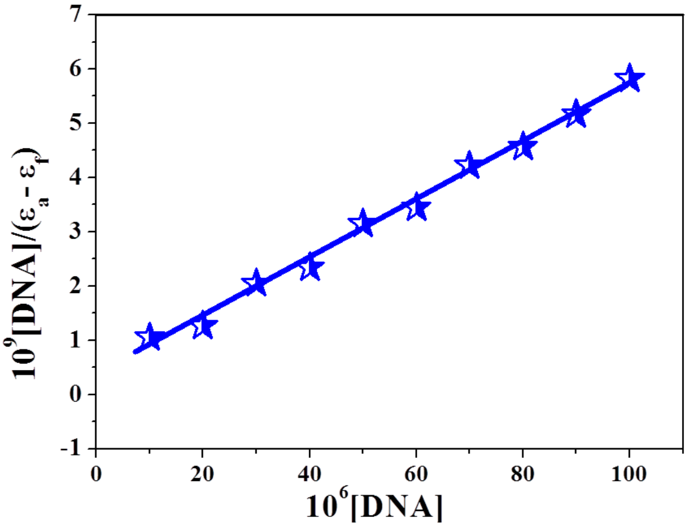

Figure S15: plot of [DNA] / (ε_a–ε_b) versus [DNA] for the interaction CT-DNA with CuL complex.

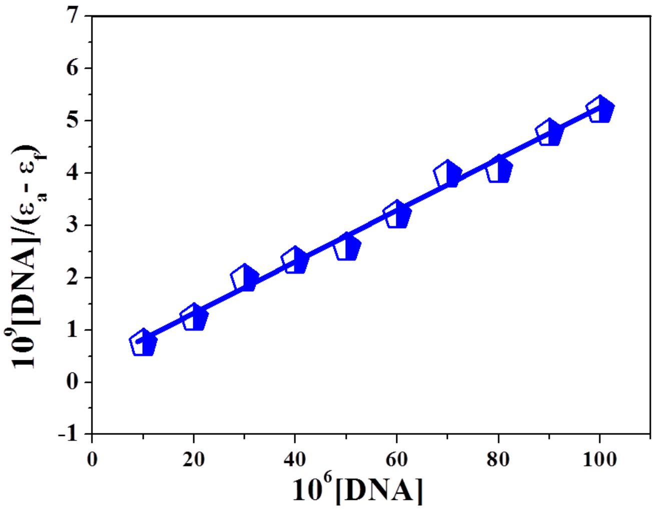

Figure S16: plot of [DNA] / (ε_a–ε_b) versus [DNA] for the interaction CT-DNA with PdL complex.

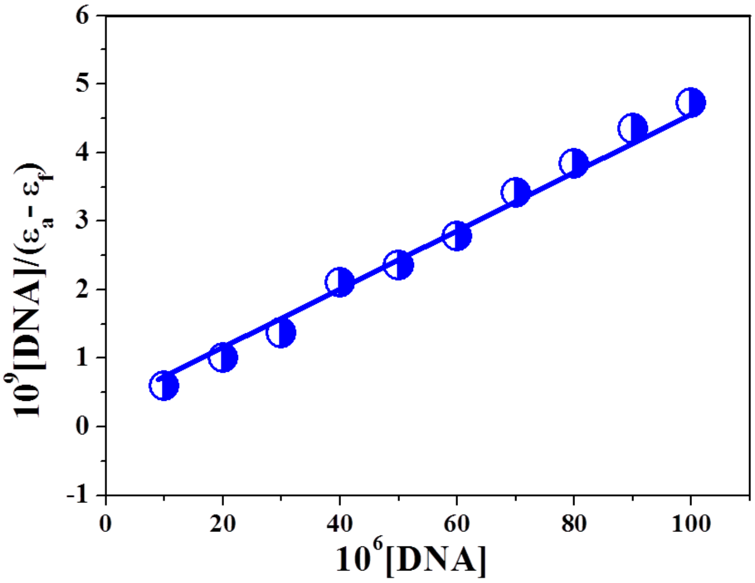

Figure S17: plot of [DNA] / (ε_a–ε_b) versus [DNA] for the interaction CT-DNA with AgL complex.

Table S1: The formation constants (K_f), the stability constant (pK) and Gibbs free energy ($\Delta G^{\neq}$) values of the studied imine complexes

Complex	Type of complex	K_f	pK	$\Delta G^{\neq}$ (KJ mol^{-1})
CuL	1:1	7.36 $X10^4$	4.87	-27.77
PdL	1:1	6.11 $X10^4$	4.79	-27.30
AgL	1:1	4.39 $X10^4$	4.64	-26.49

Table S2: The electronic spectral measurements of the prepared H_2L ligand and its complexes in DMF at 298 K against DMF as a blank.

Imine ligand and its complexes	λ_{max} (nm)	ε (dm^3 mol^{-1} mm^{-1})	Assignment
H_2L	239	883.84	π→π
	252	893.94	π→π*
	316	772.73	n→π*
	355	774.24	Intra ligand band
CuL	239	3866.00	π→π*
	252	3934.00	π→π*
	354	3480.00	n→π*
	477	3800.00	LMCT
	560	260.00	d-d
PdL	239	3360.00	π→π*
	252	3440.00	π→π*
	360	2980.00	n→π*
	430	3160.00	LMCT
	450	3140.00	and MLCT
AgL	239	3880.00	π→π*
	252	3940.00	π→π*
	305	3500.00	n→π*
	354	3480.00	LMCT
	417	3180.00	MLCT

Table S3: The natural charges and electronic configuration of the studied metals ions in the studied complexes.

Complexes	Natural charges	Core	natural population`			electronic configuration
			Valence	Rydberg	Total	
PdL	0.5572	35.976	9.443	0.0237	45.443	[Core] $5s^{0.24}4d^{8.67}\ 5p^{0.02}6p^{0.53}$
AgL	0.7238	35.989	10.277	0.0091	46.276	[Core] $5s^{0.17}4d^{9.82}5d^{0.21}6p^{0.09}$
CuL	0.935	17.980	10.070	0.051	28.065	[Core] $4s^{0.29}3d^{9.24}4p^{0.53}4d^{0.01}5p^{0.01}$

Table S4: The neutral charge on the active atom of the H_2L ligand and its complexes.

Center	H_2L	PdL	AgL	CuL
N8	-0.377	-0.458	-0.496	-0.553
O24	-0.526	-0.620	-0.762	-0.485
N10	-0.388	-0.478	-0.489	-0.538
O25	-0.563	-0.541	-0.719	-0.668

Table S5: The hyperpolarizabilities and polarizabilities of the titled compounds and Urea.

Property	Urea	H_2L	PdL	AgL	CuL
μ_x		-1.3935	-8.316	-4.933	3.638
μ_y		-7.8922	1.377	-10.975	3.373
μ_z		-1.3763	0.831	3.086	3.312
μ_{1D}	1.3197	8.1316	8.471	12.421	5.965
α_{xx}		-135.45	-271.22	-328.91	-216.73
α_{xy}		-6.995	18.70	12.75	-2.93
α_{yy}		-194.61	-263.72	3.45	-221.97
α_{zz}		-199.64	-346.84	-22.32	-263.4
α_{xz}		2.07	-8.58	15.89	-5.808
α_{yz}		8.54	8.13	-7.79	-4.07
$(\alpha)_1$ a.u.		-176.57	-293.93	-115.93	-234.03
$(\alpha)_1$ esu		-2.616 x10^{-23}	-4.356 x10^{-23}	-1.718 x10^{-23}	-3.468 x10^{-23}
$\Delta\alpha$ a.u.		87.43	112.62	452.91	62.62
$\Delta\alpha$ esu		1.2957 x10^{-23}	1.6659x10^{-23}	6.712 x10^{-23}	9.280 x10^{-24}
β_{xxx}		-156.99	-325.50	40.26	97.39
β_{xxy}		-166.18	-202.98	-284.40	-29.60
β_{xyy}		-5.97	12.92	-11.30	36.8
β_{yyy}		-93.06	-70.85	-301.71	132.5
β_{xxz}		24.76	7.40	18.51	5.34
β_{xyz}		3.18	-17.88	26.92	-20.9
β_{yyz}		-19.13	13.35	23.49	30.6
β_{xzz}		63.09	-51.81	-5.29	41.6
β_{yzz}		-36.27	-110.31	-153.05	24.6
β_{zzz}		14.04	24.99	-96.09	24.6
β_1 a.u		312.55	531.45	741.51	225.44
β esu	0.194x10^{-30}	2.71 x10^{-30}	4.591 x10^{-30}	6.41 x10^{-30}	1.947 x10^{-30}

Table S6: Cytotoxic activity (IC_{50}) in µM of the prepared compounds against Hep-G2 (hepatocellular carcinoma cell line), MCF-7 cell line (breast carcinoma cell line) and HCT-116 (colon carcinoma cell line)

Compouds	HCT-116 cell line	Hep-G2 cell line	MCF-7 cell line
H_2L	205	130.3	111.3
CuL	40.4	30.2	18.9
PdL	49.5	40.1	35.2
AgL	58.8	48.5	40.1
Vinblastine standard	13.56	7.89	4.44
